# Scabinol for Dog-mange

**Published:** 1898-09

**Authors:** 


Scabinol for Dog-mange. The following composition, named scabinol, Issleit (*Vet. Record*) recommends as a cure for mange in dogs: Soft-soap, 4 parts; betanaphtol, 1 part; storax, 2 parts; tobacco extract, 3 parts. Apply for three days, covering not more than one-third of the body at one time. Then wash the whole body with a solution of one teaspoonful of the scabinol in one quart of water.

